# Effective multidimensional treatment identification of different chemical fertilizers: Response of insect dynamics and rice production

**DOI:** 10.1016/j.heliyon.2024.e32567

**Published:** 2024-06-06

**Authors:** Sanjida Akter, Tapon Kumar Roy, Md Mozammel Haque, Zakaria Alam

**Affiliations:** a Entomology Division, Bangladesh Rice Research Institute, Gazipur, 1701, Bangladesh; b Soil Science Division, Bangladesh Rice Research Institute, Gazipur, 1701, Bangladesh; c Tuber Crops Research Centre, Bangladesh Agricultural Research Institute, Gazipur, 1701, Bangladesh

**Keywords:** *Oryza sativa*, Insect-pest, Natural enemies, Grain yield, Effective multidimensional treatment (EMT), Factor analysis, Selection differential, MGIDI

## Abstract

Effective management of fertilizers is essential in influencing the prevalence of insects in rice (*Oryza sativa* L.) fields. Over two years (2019-20 and 2020-21), an experiment conducted at Bangladesh Rice Research Institute (BRRI), Habiganj, during the boro season aimed to identify the most effective multidimensional treatment (EMT) by testing various combinations of chemical fertilizers and its effect on rice insects. The goal was to optimize rice grain yield while minimizing harmful insect infestation and supporting natural enemies. Eight different chemical fertilizer applications were used as follows: T1 contained a full mix of nitrogen (N), phosphorus (P), potassium (K), and sulfur (S); T2 had PKS but lacked N; T3 had NKS but lacked P; T4 had NPS but lacked K; T5 had NPK but lacked S; T6 had KS but lacked N and P; T7 had PS but lacked N and K; and T8 lacked all four elements - N, P, K, and S.

The relationship between the dynamics of harmful insects and natural enemies was highly positively correlated (*r* = 0.72 to 0.97). In two consecutive growing years, the 2020-21 season exhibited notably higher counts of harmful insects, with Rice Leafroller (RLR) dominating in the booting stage and White Backed Planthopper (WBPH) in mid-tillering, while Green Mirid Bug (GMB) prevailed among natural enemies across both stages, surpassing insect pest counts, notably GMB, Lady bird beetle (LBB), Carabid beetle (CDB), and Staphylinid (STD). However, the yield was notably higher in the 2019-20 growing season despite these pest pressures. Throughout the mid-tillering and booting stages, T1 consistently exhibited the highest average populations of harmful insects and natural enemies, while T7 demonstrated the lowest count of harmful insects, followed by T2 at both growth stages. Additionally, the highest grain yield (GY) was consistently recorded in T1, followed by T5, T6, and T3, with yields of 7.98 t/ha, 7.63 t/ha, 7.38 t/ha, and 7.33 t/ha, respectively. In both stages, beneficial insects prevailed over harmful ones in all fertilizer applications, with significant declines noted in T2 and T7. Factor analysis showed successful selection for EMT in the MGIDI index for all variables except INT and GY during the 2019-20 season, with selection differentials (SD) ranging from −0.10 to 8.29. However, in 2020-21, selection was achieved for all variables with SD ranging from 0.37 to 6.08. According to the MGIDI index, the top-ranked EMTs were identified as T4 and T3 for the 2019-20 period, and T3 and T5 for the 2020-21 period. The EMT shared in both years, T3, proved effective because of its positive impact on enhancing natural enemies throughout both periods (with SD ranging from 4.76 to 8.29 for 2019-20 and 3.03 to 6.08 for 2020-21), and its notable contribution to rice grain yield (SD = 0.37) in 2020-21. This study uniquely integrates EMT to optimize rice grain yield while simultaneously managing harmful insect infestations and supporting natural enemies, addressing a critical need in sustainable rice cultivation. The suggestion is to give preference to fertilizer application T3, which omits P but contains N and K, to improve rice grain yield and boost natural enemies, thereby reducing harmful insect infestation. Moreover, future investigations should concentrate on refining fertilizer blends to strike a harmony between maximizing yield and fostering ecological robustness in rice cultivation.

## 1 Introduction

Cultivation of rice (*Oryza sativa* L.) is notably concentrated in Asia, with over 90 % of global rice production occurring in this region [1]. The primary rice-producing nations, numbering the top ten, are exclusively Asian. This cereal grain holds a significant dietary role for more than 60 % of the world's population, particularly in Asia, where it contributes substantially to daily caloric intake [2]. Asia takes charge of 90 % of global rice production, with the epicenter of cultivation situated in countries in southern and southeastern Asia [3]. Over the past few decades, Bangladesh has witnessed significant increase in total rice production. Historical data [4] indicates a remarkable increase in rice production, with the adoption of high-yielding varieties, improved agricultural practices.

In Bangladesh, agriculture plays a pivotal role in driving economic growth, particularly through the cultivation of three main rice crops: Aus, Aman, and Boro, each grown in distinct seasons. Among these, Boro stands out as the dominant contributor to the nation's rice production, accounting for over 50 %, while Aus contributes a minor fraction, less than 10 %. Over time, the cultivation of Boro rice has significantly bolstered Bangladesh's overall rice yield, as consistently observed in agricultural data [4]. The reliance on Boro rice becomes increasingly crucial as the country aims to meet the substantial demand for rice, projected to reach 44.6 million tons (MT) of milled rice by 2050 to sustain a population of approximately 215.4 million [5]. However, rapid industrialization and infrastructure expansion compound this challenge by diminishing land availability. To address this, a vital strategy involves implementing balanced fertilization methods, combining both inorganic and organic nutrient sources, while improving agronomic practices. This approach becomes essential in efforts to boost rice productivity and maintain soil quality [[6], [7], [8]].

Despite the prevalent use of chemical fertilizers in Bangladesh, farmers exhibit a preference for higher urea application compared to other fertilizers [9]. However, this farming practice has significant implications for the delicate ecological balance within rice fields, affecting not only the rice plants but also the diverse insect populations that inhabit this agricultural system. Rice, known for its exceptional nutritional value compared to other cereal crops, serves as an ideal habitat for over 800 insect species [10]. In tropical Asia, more than 100 insect species thrive within rice ecosystems. Bangladesh specifically identifies 232 rice insect pests, contributing to the global count of over 100 insects recognized as pests in rice production worldwide. Interestingly, the rice ecosystem in Bangladesh supports 375 beneficial arthropod species [11], but only around 20 to 33 species have considerable economic impacts. Insect pests lead to an average yield loss of 20 % in Asia, where more than 90 % of the world's rice is grown [12].

While managing nutrients remains critical in rice production, its influence extends to how rice plants respond to insect pests. Recognizing the positive connections between nutrients and insects can provide guidance for optimizing the overall function of the agricultural ecosystem [13]. Rice crops face various insects such as the brown plant hopper, green leafhopper, stem borer, rice leafroller, grasshopper, and case worm. Within this context, beneficial insects, classified as predators or natural enemies, hold a crucial role in engaging with and controlling these pests to maintain them at manageable levels.

In recent times, novel principles, technologies, and pest management strategies have emerged, with 'green plant protection' gaining widespread acceptance in China [14]. Another set of strategies, falling under ecological control practices, aims to reduce insecticide use, with ecological engineering being a notable approach. Understanding the impact of fertilizers on rice field insects and their predators in Bangladesh is incredibly significant, as it profoundly affects agricultural output and the environment. Exploring the complex connections between standard fertilizer use, pests, natural predators, and rice yield is crucial for effective pest management in Bangladesh's unique farming environment.

The introduction of the innovative concept "effective multidimensional treatment" (EMT) for addressing multiple parameters explores these complex relationships to improve biological output as intended. When selecting genotypes, researchers typically focus on specific combinations of plant traits to cultivate an ideal plant for a particular environment, maximizing output or yield. This targeted genotype is termed an “plant ideotype”, a concept pioneered by Donald in 1968 [15] in wheat genotype selection based on phenotypic performance. The core idea behind ideotype design is to enhance crop performance by selecting genotypes that exhibit multiple desirable traits simultaneously. Building on the advancement of crop modeling approaches, various effective plant genotype selection indices have been developed, including the factor analysis and ideotype-design (FAI-BLUP) index by Rocha et al. [16] and the Smith-Hazel (SH) index by Hazel [17] and Smith [18], utilized in breeding programs [[19], [20], [21]]. Among these ideotype concepts, the SH index is favored by plant breeders for selecting superior genotypes based on multiple traits [19]. However, the SH index, which involves inverting a phenotypic covariance matrix, is prone to multicollinearity issues when assessing multiple traits [22]. This susceptibility to multicollinearity can lead to poorly conditioned matrices, resulting in biased index coefficients and affecting the accuracy of selection differential (SD) estimations. In addition to dealing with multicollinearity, researchers often encounter the difficulty of assigning economic value to traits, requiring the transformation of traits into practical economic weightings [23].

In light of this, the mixed effect model is commonly used in selection processes to determine predicted mean values of study parameters and improve selection efficiency [[24], [25], [26], [27]]. To achieve this, best linear unbiased prediction methods have been employed as effective selection models [20]. A recently introduced approach, the multi-trait genotype–ideotype distance index (MGIDI), is tailored for genotype selection based on predicted breeding values by incorporating information from multiple traits [22,28]. Identifying EMT is a novel concept that supersedes the term "plant ideotype" in MGIDI. While traditional ranking methods like Fisher's LSD or Tukey's HSD in ANOVA evaluate effective treatments based on single performance parameter, the MGIDI index can detect EMT by considering performances across multiple parameters under study. Moreover, MGIDI addresses concerns about collinearity in assessing SDs for predicting EMT performance by conducting factor analysis [22]. This involves utilizing varimax rotation criteria [29] for rotational analysis and estimating factor loadings of study parameters after flexible rescaling according to researchers' preferences. This technique has been successfully applied in various crop breeding programs for genotype selection, such as wheat [30], rice [31] and maize [32]. Jalalifar et al. [33] have recently introduced an advanced tool that enables breeders to efficiently select the most desirable genotypes with resistance to leaf blast in rice considering the interaction between outcome across multiple years. Consequently, in present study, EMT is anticipated to contribute to increased rice yields and has the potential to alleviate insect pest pressures, thereby reducing the incidence of pest outbreaks and minimizing yield losses. It can also promote the proliferation of beneficial insect populations, including predators and natural enemies of insect pests, thereby enhancing biological pest control mechanisms and reducing reliance on chemical pesticides. The objective of the current investigation was to identify EMT and assess the impact of chemical fertilizer combinations on insect dynamics in rice production.

## 2 Materials and methods

### 2.1 Experimental site

**Fig. 1:**
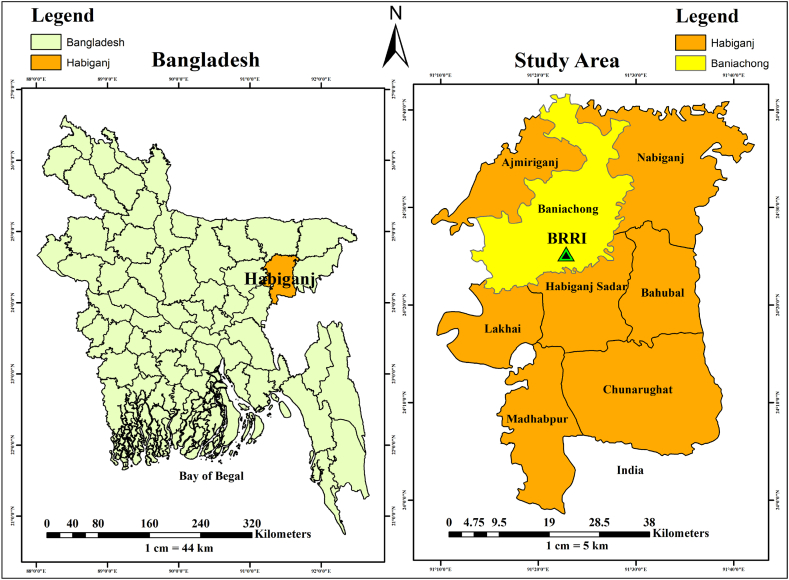
Location of study area.

The investigation was conducted within the irrigated rice field ecosystem situated at the regional station of the Bangladesh Rice Research Institute (BRRI) in Baniachong, Habiganj, Sylhet, Bangladesh (Latitude 24°22′59.9″N, Longitude 91°25′00.1″E) (Fig. 1), with an elevation 12 m above sea level. Geographically, the site is located within the old Meghna estuary floodplain zone, falling under Agro-Ecological Zone-19. The experimental site's soil composition is characterized as clay with an acidic profile (pH 5.6), organic matter content 2.9 %, total N content 0.9 %, available P 20 mg kg^−1^, available K 50 mg kg^−1^, available S 24 mg kg^−1^ and available Zn 1.0 mg kg^−1^. Fertility level of soil is medium. The soil of experimental site is classified as Mollic Gleysols [34]. The experiment was conducted during the boro season spanning from December to April (transplanting to harvesting) for two consecutive years (2019–2020 and 2020–2021). The weather data of experimental site during 2019-20 and 2020-21 was recorded and presented in Table 1.

**Table 1:** Mean temperature, precipitation, humidity and sunshine statistics during growing period of 2019-20 and 2020-21 in Habiganj.

Months	2019–20			2020–21		
	Temperature (⁰C)	Precipitation (mm)	Humidity (%)	Temperature (⁰C)	Precipitation (mm)	Humidity (%)
**November**	26.20	68.77	65.61	25.56	0.00	74.64
**December**	20.60	80.13	68.14	22.03	1.30	80.90
**January**	18.29	0.54	78.87	20.36	78.10	79.00
**February**	20.25	0.00	67.76	22.40	62.23	69.00
**March**	24.85	0.00	63.23	26.34	50.81	60.00
**April**	25.99	2.99	67.30	28.98	61.13	68.53

### 2.2 Treatments, design and fertilization

Eight different chemical fertilizer combinations were applied as follows: T1 consisted of a complete mix of nitrogen (N), phosphorus (P), potassium (K), and sulfur (S) (NPKS/complete); T2 included PKS but excluded N (PKS/-N); T3 included NKS but excluded P (NKS/-P); T4 included NPS but excluded K (NPS/-K); T5 included NPK but excluded S (NPK/-S); T6 included KS but excluded N and P (KS/-NP); T7 included PS but excluded N and K (PS/-NK); and T8 excluded all four elements - N, P, K, and S (all missing/-NPKS). The application rates for NPKS were 13.8-2.0-8.0–1.0 g/m^2^ (138-20-80-10 kg/ha) respectively and applied as urea, triple super phosphate, muriate of potash and gypsum respectively [35]. Nitrogen was administered in three equal splits at the basal (during land preparation), maximum tillering, and panicle initiation stages (excluding the N omission plot), phosphorus as a basal application (excluding the P omission plot), and potassium at both the basal and panicle initiation stages (excluding the K omission plot). Treatments were arranged in a completely randomized block design with three replications. Unit plot size was 5- × 3-m. The cropping pattern was Boro-Fallow-Fallow, with BRRI dhan92 serving as the test crop. Forty-day-old 2 seedlings hill^−1^ were transplanted at 20- × 20-cm spacing during first week of December and harvested in mid of April in both the years.

### 2.3 Crop management

Rigorous implementation of essential irrigation and management techniques was undertaken according to BRRI [36]. Flood irrigation and tilling were conducted following the traditional tillage approach for cultivating puddled transplanted rice. A shallow layer of water was sustained, kept approximately 5–7 cm from the soil surface, until the soft dough stage (three weeks prior to rice harvesting) of plant growth stage. All other cultural practices such as hand weeding adhered to standard recommendations with no insecticide usage in the experimental field.

### 2.4 Data collection

Insect data were collected through 20 complete sweeps conducted in each plot and direct count of all the harmful insect and beneficial insect in both mid-tillering (mid-January) and booting stage (first week of February). Insects were identified by their morphological appearance. Harmful insects including the brown plant hopper (BPH) (*Nilaparvata lugens*), white-backed plant hopper (WBPH) (*Sogatella furcifera*), yellow stem borer (YSB) (*Scirpophaga incertulas*), rice leaf roller (RLR) (*Cnaphalocrocis medinalis*), grasshopper (GH) (*Hieroglyphus banian*), long-horned cricket (LHC) (*Euscyrtus concinnus*), green leafhopper (GLH) (*Nephotettix virescens*), case worm (CW) (*Nymphula depunctalis*), and natural enemies including ladybird beetle (LBB) (*Micraspis discolor*), carabid beetle (CDB) (*Ophonea indica*), green mirid bug (GMB) (*Cyrtorhinus lividipennis*), damselfly (Dam. Fly) (*Ceriagrion cerinorubellum*), dragonfly (Drag. Fly) (*Diplacodes trivialis* and *Brachythemis contaminata*), staphylinid beetle (STD) (*Paederus littoralis*), and spider (SPD) were recorded. Harvesting of crop was executed from a designated 5 m^2^ area centrally located within each plot at physiological maturity stage, with rice grain yield (GY) adjustments made to accommodate a 14 % moisture content on a sundry basis and yield converted into t/ha.

### 2.5 Statistical analysis

#### 2.5.1 EMT design and rescaling the parameters

To proceed selection index, the original data is needed to be rescaled. Before proceeding with the rescaling process, a Likelihood Ratio Test was carried out to see the significance of each parameter at *p* < 5 probability level with fertilizer treatments. The rescaled value for each treatment and parameter in a two-way table can be calculated using the equation for rX_*ij*_.

$$rX_{ij}=\frac{\eta_{nj}-\varphi_{nj}}{\eta_{oj}-\varphi_{oj}}\times \left(\theta_{ij}-\eta_{0j}\right)+\eta_{nj}$$

Where the new maximum and minimum values for a parameter after rescaling are denoted as η_*nj*_ and φ_*nj*_, respectively, while the original maximum and minimum values for the parameter are denoted as η_*oj*_ and φ_*oj*_, and θ_ij_ represents the original value for the *j*th parameter of the *i*th treatment. The selection of η_*nj*_ and φ_*nj*_ is determined in the following manner: when aiming for decrease selection sense in parameters, one should utilize η_*nj*_ = l and φ_*nj*_ = h, whereas for increase selection sense in parameters, η_*nj*_ = h and φ_*nj*_ = l should be employed [22]. It is important to note that there was an aim of increase selection sense for natural enemies' count (NET and NEB) and rice grain yield (GY), whereas the decrease selection sense was set for harmful insects' count (INB and INT).

For EMT selections, each parameter was analyzed using the “metan” package of R studio considering the following mixed-effect model:

y=Xb+Zu+e

where y is an $n\left[=\sum_{j=1}^{r}\;\left(gr\right)\right]\times 1$ vector of response variable, i.e. the response of the i th treatment in the j th block (i=1,2,…,g;j=1,2, $\ldots ,r;y={\left[y_{11},y_{12},\ldots ,y_{gr}\right]}^{\prime });b$ is an 1×r vector of unknown and unobservable fixed effects of block $b={\left[\gamma_{1},\gamma_{2},\ldots ,\gamma_{r}\right]}^{\prime }$; u is an m[= 1×g] vector of unknown and unobservable random effects of treatment $u={\left[\alpha_{1},\alpha_{2},\ldots ,\alpha_{g}\right]}^{\prime };X$ is an n×r design matrix of 0s and 1s relating y to b;Z is an n×m design matrix of 0s and 1s relating y to u; and e is an n×1 vector of random errors $e={\left[y_{11},y_{12},\ldots ,y_{gr}\right]}^{\prime }$.

#### 2.5.2 MGIDI index

The MGIDI was calculated by determining the Euclidean distance between the scores of different treatments [37]. The ideal score of treatments was computed with the following equation-

$${MGIDI}_{i}={\left[\sum_{j=1}^{f}{\left(\gamma_{ij}-\gamma_{j}\right)}^{2}\right]}^{0.5}$$

Where, MGIDI_i_ denotes the distance index of multi-trait genotype-ideotype pertaining to the *i*th treatment. In this context, the score of the *i*th treatment in the *j*th factor, represented by γ_*ij*_, holds significance. The variables *i* and *j* range from 1 to the number of treatment (*t*) and factors (*f*), respectively. Furthermore, γ_*j*_ signifies the *j*th score of the EMT. The treatment with the lowest MGIDI is then closer to the EMT and therefore presents desired values for all parameters.

#### 2.5.3 Factor analysis

Factor analysis was computed with X to account for data correlation and reduce dimensionality as follows [22].

X=μ+Lf+ε

Where, the vector of original observations, denoted by X, consists of *p* × 1 elements. The vector μ represents the standardized means and also has *p* × 1 dimensions. The matrix L, with dimensions' *p* × *f*, contains the factorial loadings. The vector *f*, with *p* × 1 dimensions, represents the common factors. The vector ε, also with *p* × 1 dimensions, represents the residuals. Here, *p* and *f* denote the number of parameters and common factors retained, respectively. The correlation matrix of rX_ij_ provides the eigenvalues and eigenvectors. The initial loadings are obtained by considering only factors with eigenvalues greater than one. Subsequently, the varimax [29] rotation criteria is utilized for the analytic rotation and estimation of the final loadings. The scores are then obtained as follows:

$$F=Z{\left(A^{T}R^{-1}\right)}^{T}$$

where F represents a *t* × *f* matrix containing the scores obtained from factorial analysis; Z denotes a *t* × *p* matrix consisting of the (rescaled) standardized means; A refers to a *p* × f matrix representing the canonical loadings; and R signifies a *p* × *p* correlation matrix that captures the relationships between the parameters. Here, *t*, *f,* and *p* are used to denote the number of treatments, factors retained, and analyzed parameters, respectively.

The Pearson's correlation of coefficient matrix with heatmap for all the rice insect species were analyzed using ‘metan’ package of R software. The pooled ANOVA of two rice growing seasons was conducted to assess the effect of fertilizer treatments on insect count (harmful and beneficial) and rice grain yield. The means of different treatments for insect count and grain yield were compared using Least Significant Different Test (LSD) at p < 0.05 with ‘agricolae’ package of R software. All graphs were prepared using Microsoft excel and R software. For selecting EMT, the multi trait genotype ideotype index (MGIDI) was analyzed using ‘metan’ package of R software [38].

## 3 Results

### 3.1 Insect species and their natural enemy counts in rice field

**Fig. 2:**
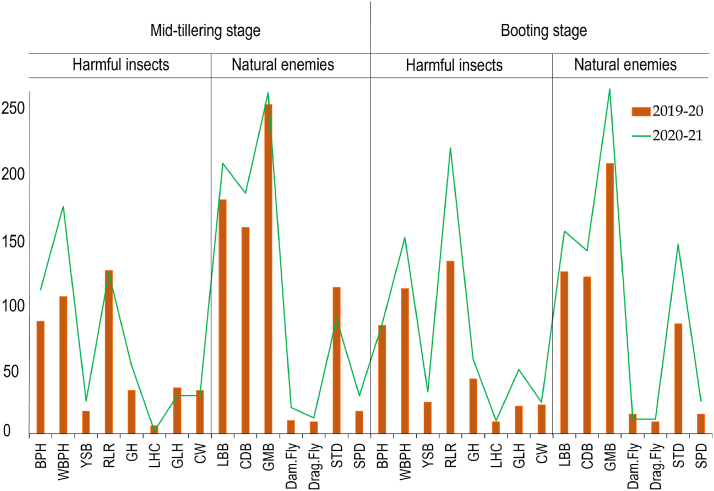
The total number of different insect pest and natural enemies collected in the experimental field during 2019-20 and 2020-21. Where, the harmful insects including the brown plant hopper (BPH), white-backed plant hopper (WBPH), yellow stem borer (YSB), rice leaf roller (RLR), grasshopper (GH), long-horned cricket (LHC), green leafhopper (GLH), case worm (CW), and natural enemies including ladybird beetle (LBB), carabid beetle (CDB), green mirid bug (GMB), damselfly (Dam. Fly), dragonfly (Drag. Fly), staphylinid beetle (STD), and spider (SPD). (For interpretation of the references to color in this figure legend, the reader is referred to the Web version of this article.)

Fig. 2 illustrates various harmful insects and natural enemies counted in the experimental field of two consecutive growing years under investigation at mid-tillering and booting stage of rice. In terms of 2019-20, the total RLR count was highest in both mid-tillering stage and booting stage amongst harmful insects, while GMB was found highest amongst natural enemies in both stages of rice. In terms of 2020-21, WBPH was highest in mid-tillering stage amongst harmful insect categories. On the other hand, RLR was highest in booting stage. In case of natural enemies, GMB was highest in both tillering and booting stage. The count of both insect categories, as well as their natural enemies, was notably greater during the 2020-21 growing season in comparison to the preceding year of 2019-20. Additionally, the count of natural enemies was higher than insect pests in both rice stages, especially GMB, LBB, CDB and STD.

### 3.2 Coefficient of correlation between insect species and their natural enemies

**Fig. 3:**
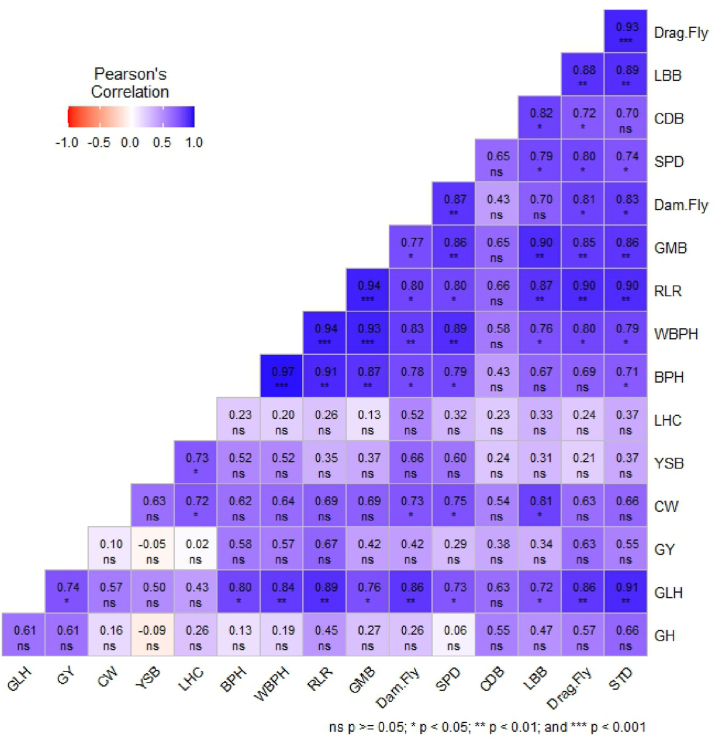
Pearson's coefficient of correlations among different rice insect pests. Where, the harmful insects including the brown plant hopper (BPH), white-backed plant hopper (WBPH), yellow stem borer (YSB), rice leaf roller (RLR), grasshopper (GH), long-horned cricket (LHC), green leafhopper (GLH), case worm (CW), and natural enemies including ladybird beetle (LBB), carabid beetle (CDB), green mirid bug (GMB), damselfly (Dam. Fly), dragonfly (Drag. Fly), staphylinid beetle (STD), and spider (SPD). (For interpretation of the references to color in this figure legend, the reader is referred to the Web version of this article.)

Fig. 3 illustrates the correlations among insect pests during rice cultivation in 2019-20 and 2020-21 season. A high positive correlation was found between WBPH and BPH (*r* = 0.97), RLR and WBPH (*r* = 0.94), GMB and WBPH (*r* = 93), GMB and RLR (*r* = 94), Drag. Fly and STD (*r* = 0.93). There was no significant negative correlation observed between insect pests of rice.

### 3.3 Estimation of variations in fertilizer treatments and growing years for harmful insect, natural enemy count and grain yield

Fertilizer treatments and seasonal years significantly affected the counts of harmful insects and natural enemies during both mid-tillering and booting stages, with a probability level of p ≤ 0.05 (Table 2). Furthermore, fertilizer treatments and growing years significantly affected the grain yield of rice. However, no significant interaction effect was observed between seasonal years and fertilizer treatments for rice insects, except for the grain yield.

**Table 2:** Pooled ANOVA of two rice growing seasons for eight fertilizer treatments on insect (harmful insects and natural enemies) count at both mid tillering and booting stage and grain yield of rice cultivation.

Source of variations	DF	Mean sum of squares				
		INT	INB	NET	NEB	GY (t/ha)
**Fertilizer treatment (T)**	7	852.6***	913***	1029.6***	1163.1***	3.39***
**Years (Y)**	1	253.4*	717.8***	78.9*	869.8***	4.48***
**T × Y**	7	54.6^NS^	52.7 ^NS^	33.9 ^NS^	59.9 ^NS^	0.12***
**Residuals**	34	59.3	39	15	50.8	0.014

DF = degrees of freedom, *** = 0.1 % level of significant, * = 5 % level of significant, NS = non-significant. INT = number of harmful insects at mid-tillering stage, INB = number of harmful insects at booting stage, NET = number of natural enemies at mid-tillering stage, NEB = number of natural enemies at booting stage, GY = rice grain yield.

### 3.4 Effect of fertilizer treatments and growing years on insects and rice grain yield

The effect of fertilizer treatments in the insect count and rice grain yield are presented in Table 3. The highest average populations of harmful insects and natural enemies were observed in the complete fertilizer application (NPKS) during both the mid-tillering and booting stages (47.33, 50.50, 55.83, and 54.17, respectively). During the mid-tillering stage, PS recorded the lowest count of harmful insects (9.83), followed by PKS (10.83) and KS (14.33). During this stage, natural enemies exhibited the lowest count in KS (17.33), followed by PS (20.17) and PKS (24.83). Transitioning to the booting stage, PS exhibited the lowest population of harmful insects (9.17), followed by PKS (15.50) and KS (18.50).

**Table 3:** Average grain yield, insect count in different fertilizer applications during 2019-20 and 2020-21 seasons.

Fertilizer treatments	INT	INB	NET	NEB	GY (t/ha)
T1 (PKS)	47.33a	50.50a	55.83a	54.17a	7.98a
T2 (PKS)	10.83d	15.50cd	24.83d	17.67c	6.37f
T3 (NKS)	24.50b	23.50b	41.50b	30.33b	7.33c
T4 (NPS)	21.83BCE	20.83BCE	40.17b	36.33b	6.67e
T5 (NPK)	18.67b-d	22.17BCE	31.50c	29.00b	7.63b
T6 (KS)	14.33cd	18.50BCE	17.33e	13.50c	7.38c
T7 (PS)	9.83d	9.17d	20.17e	12.17c	6.94d
T8 (all missing)	18.00b-d	17.25BCE	24.75d	32.75b	5.82g
CV (%)	37.43	28.43	12.22	25.08	1.67

INT = number of harmful insects at mid-tillering stage, INB = number of harmful insects at booting stage, NET = number of natural enemies at mid-tillering stage, NEB = number of natural enemies at booting stage, GY = rice grain yield, CV = coefficient of variation. Values in a column with different letter(s) are significantly different at p ≤ 0.05 applying LSD test.

**Fig. 4:**
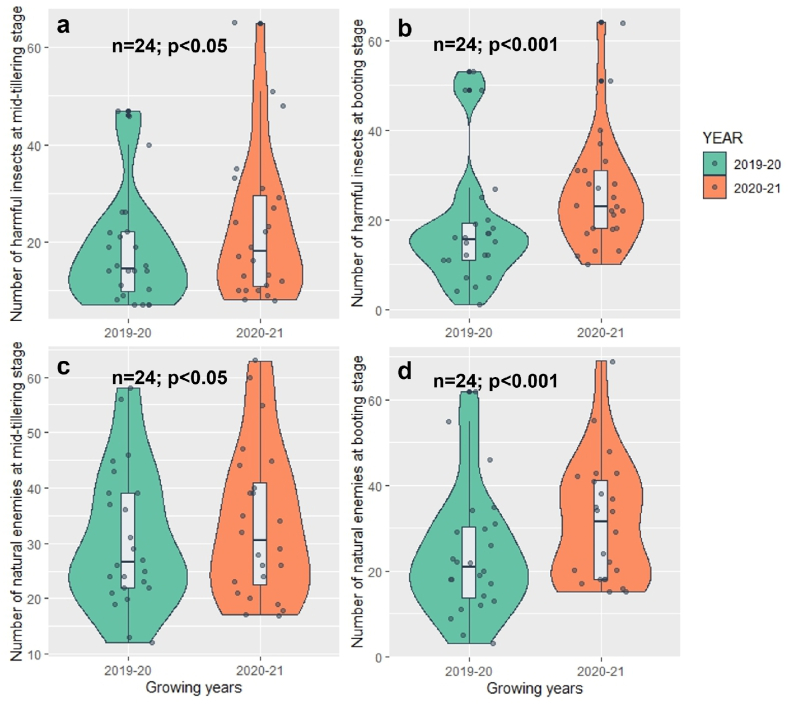
Average count of harmful insects (a and b) and natural enemies (c and d) in two rice growing years (2019-20 and 2020-21). ^n^ sample size, ^p^ significance level.

**Fig. 5:**
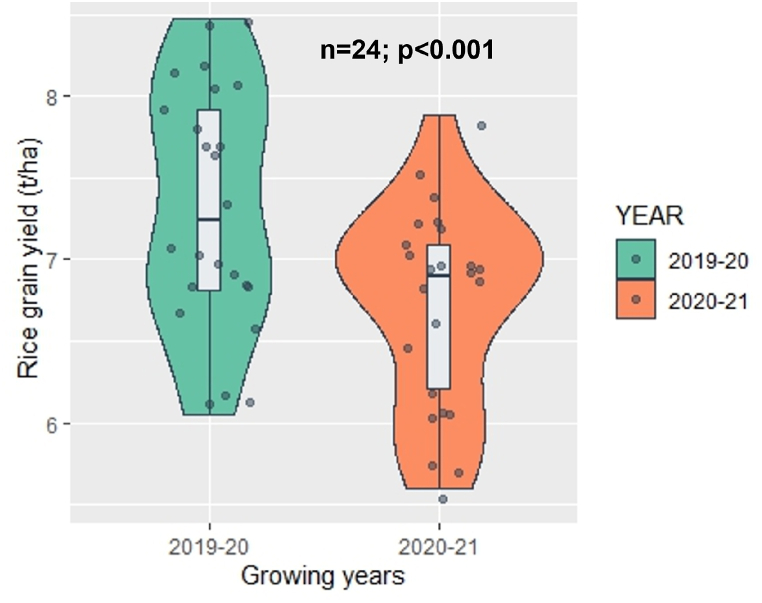
The average grain yield (t/ha) of two growing years (2019-20 and 2020-21). ^n^ sample size, ^p^ significance level.

In terms of natural enemies during the booting stage, PS recorded the lowest count (12.17), followed by KS (13.50) and PKS (17.67). As for grain yield, NPKS demonstrated the highest yield (7.98 t/ha), followed by NPK (7.63 t/ha) and KS (7.38 t/ha). The lowest yields were observed in the treatment of all missing fertilizers (-NPKS) (5.82 t/ha), followed by PKS (6.37 t/ha) and NPS (6.67 t/ha) (Table 3). In Fig. 4, it is found that both the harmful insects (Fig. 4a and b) and natural enemies (Fig. 4c and d) during mid tillering and booting stages were significantly higher in 2020-21 growing season than 2019-20 season. In Fig. 5, result showed that the rice grain yield was significantly higher during 2019-20 growing season (7.5 t/ha) than that of 2020-21 season (6.9 t/ha).

### 3.5 Relationship of harmful insect and their natural enemies

**Fig. 6:**
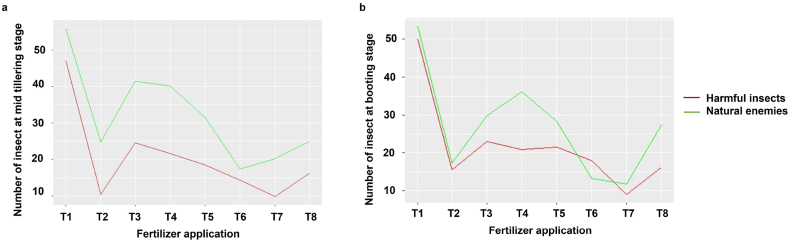
Comparison of harmful insects and natural enemies' response in different fertilizer treatment during the mid-tillering (a) and booting stages (b) at rice cultivation during two consecutive growing years at Habiganj, Bangladesh, where, T1 = NPKS (Complete), T2 = PKS (-N), T3 = NKS (-P), T4 = NPS (-K), T5 = NPK (-S), T6 = KS (-NP), T7 = PS (-NK), and T8 = all missing (-NPKS).

In Fig. 6a, the count of natural enemies surpassed that of harmful insects in all the treatments during the mid-tillering stage. Notably, in PKS and PS, there was a robust decrease in natural enemies and harmful insects. Moving on to Fig. 6b, it was also observed that the count of harmful insects was lower than that of natural enemies in different fertilizer applications, with the exception of KS. There was a sharp decrease found also in PKS and PS for both harmful insect and natural enemies in booting stage. In both the growth stages, highest natural enemies found in NPKS fertilizer application, followed by NKS (Fig. 6a and b).

### 3.6 Selection of EMT using MGIDI

In factor analysis, the sense of selection for EMT in MGIDI index was successfully achieved for all the studied variables except INT and GY for 2019-20 season, which are two weaknesses for EMT during 2019-20 season. The selection differential (SD) was ranged from 0.10 to 8.29 (Table 4). In 2020-21, the selection sense was achieved for all studied variables and the selection differential of EMT ranged from 0.37 to 6.08 (Table 4).

**Table 4:** Factor analysis for five studied variables with their BLUP values and the selection differential percentage of EMT obtained using MGIDI selection index.

Variables	Selection sense	2019–20				2020–21			
		X_o_	X_s_	SD	goal	X_o_	X_s_	SD	goal
INB	Decrease	18.30	17.20	−1.05	100	26.00	28.10	2.06	0
INT	Decrease	18.30	20.70	2.41	0	22.70	26.20	3.58	0
NET	Increase	30.70	39.00	8.29	100	33.40	39.50	6.08	100
NEB	Increase	23.90	28.70	4.76	100	31.30	34.30	3.03	100
GY	Increase	7.32	7.23	−0.10	0	6.72	7.09	0.37	100

X_o_ = observed mean, X_s_ = predicted mean, SD = selection differential, INB = harmful insects at mid-tillering stage, INT = harmful insects at booting stages, NEB = natural insects at booting stages and NET = natural enemies at mid-tillering stage.

**Fig. 7:**
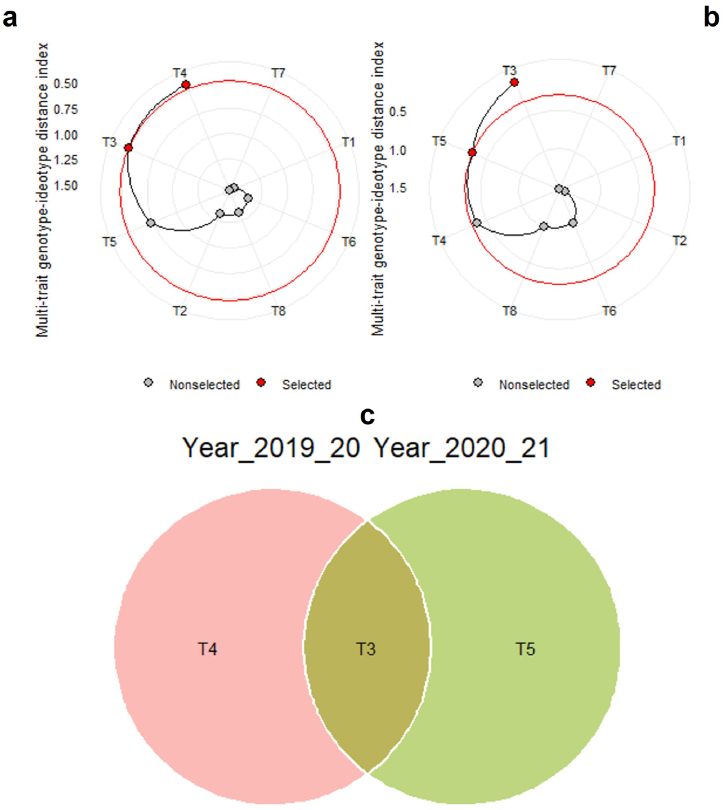
Identifying EMT and performance ranking of fertilizer treatments based on insect counts (harmful insects and natural enemies) and grain yield of rice during 2019-20 (a) and 2020-21 (b) using MGIDI and a Venn diagram (c) for selecting common EMT of two consecutive years' selection. Where, T1 = NPKS (Complete), T2 = PKS (-N), T3 = NKS (-P), T4 = NPS (-K), T5 = NPK (-S), T6 = KS (-NP), T7 = PS (-NK), and T8 = all missing (-NPKS).

The fertilizer treatments were organized in a descending manner in accordance with the MGIDI index, with the treatments possessing the highest index value positioned at the center, while those with the lowest index value are placed on the outermost part of the circle (Fig. 7a and b). The dots colored in red indicated the selection of EMTs based on their MGIDI index values. The red circle in the index is cut off point for treatment selection. During the 2019-20 growing season, identified EMT was NPS, followed by NKS (Fig. 7a). In case of 2020-21 growing season, identified EMT was NKS, followed by NPK (Fig. 7b). In Venn diagram, the common EMT was NKS between both growing years (Fig. 7c). The EMT NKS consistently exhibited favorable SD for natural enemies during both time studied years, ranging from 4.76 to 8.29 for 2019-20 and 3.03 to 6.08 for 2020-21. Additionally, it notably showed a positive SD of 0.37 in relation to rice grain yield in 2020-21.

## 4 Discussion

The infestation of RLR was highest among other harmful insects in both stages (booting and tillering) of rice growth, followed by WBPH and BPH in present study (Fig. 2). In the rice fields of Bhandara, India, CBD were found to be dominant predators feeding on pests in the rice ecosystem, with the highest population of CBD predator recorded at about 56 days after transplantation [39], which in the line with present study as the CBD count was highest in mid-tillering stage in rice field for both growing seasons 2019-20 and 2020-21. The highest count for rice insect throughout the cultivation period was found for a natural enemy named GMB. The clustering of GMB with a dense population of plant hoppers is a behavioral characteristic influenced by natural regulatory processes, as GMB serves as a predator of plant hoppers (PH). The presence of natural enemy's such as SPD, GMB, and LBB is crucial in regulating PH populations within their ecosystem [[40], [41], [42]]. The decline in populations of the WBPH during crop maturation is influenced by the regulation exerted by natural enemies, as noted by Kettle et al. [43], and the changes in rice susceptibility as the plants mature, as observed by Horgan et al. [44].

Beneficial insects thrive in ecosystems rich with harmful ones [45]. This correlation is echoed in our current research, emphasizing the close link between natural enemies and the presence of pests (Fig. 3). These natural enemies, such as LBB, SPD, CDB, and Dam. Fly, target harmful insects at various life stages, from eggs to adults. For example, LBB and SPD prey on planthoppers, while CDB focus on leaf folder larvae, consuming several per day [46]. The highest abundance of SPD was noted during the tillering stage, representing predators of both larval and adult stages of SB and RLR. LBB and GMB were observed in significant numbers, primarily consuming eggs and larvae of RLR and PH [47]. Dam. fly nymphs, found in rice field water, climb rice plants to hunt PH nymphs, while adult Dam. fly seek PH insects in the rice canopy during flight [46]. Notably, Kumar et al. [42] discovered a positive link between the appearance of SPD and GMB with the presence of WBPH, reinforcing our findings.

The count of harmful insects and natural enemies fluctuated across different stages of rice growth, depending on the type of fertilizer applied. Specifically, insect counts were higher in nitrogen (N)-rich fertilizers (NPKS, NKS, NPS, and NPK) (Table 3). Lu et al. [48] emphasized the significant role of N in shaping herbivore populations. The use of N fertilizer in plants typically enhances various aspects of herbivore behavior and population dynamics, including feeding preferences, consumption rates, survival, growth, reproduction, and overall population density. In a study, the increase in N levels led to darker plant coloration due to higher chlorophyll concentration, rendering them less attractive to foraging parasitoids. Consequently, the proportion of BPH eggs parasitized decreased [49]. de Kraker [50] suggested that higher nitrogen levels in rice fields resulted in increased occurrences of RLR larvae, causing various leaf injuries. This rise in larval density was attributed to nitrogen fertilization's positive effects on egg recruitment and medium-sized larval survival. Moreover, Bala et al. [51] noted that N application alone or in high amounts increased insect populations, while the application of P and K, either separately or in combination with N, decreased population growth (Table 3). Elevated levels of K confer robust resistance against insect pests by boosting secondary compound metabolism, reducing carbohydrate accumulation, and mitigating plant damage caused by insects. Phosphorus reduces the suitability of hosts for various insect pests. Additionally, secondary macronutrients and micronutrients like Zn and S contribute to diminishing pest populations. However, P fertilization has been observed to significantly boost the population growth of BPH and YSB [52,53], which contradicts the findings of our study. Application of P alone or in combination with N and K fertilization has been noted to support moderate leafhopper populations, as reported by Kulagold et al. [54]. In our study, the highest rice grain yield was observed with the application of complete fertilizer NPKS (Table 3), consistent with the findings of Rashid et al. [53], who advocated for the application of essential nutrients in rice fields to sustain productivity. Additionally, Sun et al. [55] highlighted in their research that optimal N levels contribute to improved rice biomass, physiological aspects, and ultimately lead to increased grain yield.

The count of harmful insects and natural enemies during both the tillering and booting stages was higher in the experimental rice field of BRRI, Habiganj, in the 2020-21 season compared to the 2019-20 season (Fig. 4), as indicated by the individual insect count in the study (Fig. 2). The high temperature during the rice growing season of 2020-21 than of 2019-20 (Table 1) might be the case of high incidence of insect in 2020-21 season in both mid-tillering and booting stage. Insects are highly sensitive to temperature fluctuations, with their metabolic rate roughly doubling for every 10 °C rise in temperature [56] and temperature influences various aspects of insect physiology, including metabolism, metamorphosis, movement, and the availability of hosts, thereby impacting pest population dynamics [[57], [60]]. Moreover, warmer temperatures directly impact insect herbivores by boosting the number of generations they can complete within a single season or crop cycle [58]. In present study, rainfall until mid-tillering stage of 2019-20 season (Table 1) might have reduced the insect population in the rice field. According to Karthik et al. [59], rainfall diminishes insect growth and development by dislodging them from plants, reducing their numbers in the field, and washing away insect eggs, larvae, nymphs, and small insects [60]. The infestation of SB and RLR are favored by humid weather [61], as the prolong humid period from transplanting to tillering stage during 2020-21 (Table 1) helped to increase the infestation of RLR during tillering and booting stage.

The production of rice is susceptible to the impact of rainfall patterns, and extreme events can significantly affect yield. Such occurrences may reduce rice output by limiting the availability of N, crucial for tillering, and causing disruptions to pollination, leading to decreased panicle effectiveness per unit area and fewer filled grains per panicle [62]. During the 2020-21 growing season, significant rainfall occurred from the mid-tillering stage to the grain filling stage (Table 1), likely contributing to a yield decline compared to the 2019-20 season (Fig. 5). According to de Kraker et al. [50], balanced applications of essential nutrients and favorable weather conditions enhance the utilization efficiency of N fertilizer in rice.

The application of N-rich fertilizers (NPKS, NKS, NPK, and NPS) had a positive impact on the population dynamics of both harmful insects and natural enemies during both growth stages of rice (Fig. 6). Elevated N levels were found to promote the synthesis or accumulation of proteins, free amino acids, and sugars, potentially attracting insects, which corresponds with the findings of our study [51]. Pandey [63] reported that N application stimulates abundant crop growth, attracting pests like RLR for feeding, shelter, and egg-laying, thus making plants more vulnerable to damage. Additionally, Ghosh [64] observed a positive correlation between N application and YSB infestation, likely due to increased water content in plant tissues facilitated by N uptake. This higher water content makes plants preferred sites for YSB egg-laying and easier targets for neonate larvae after hatching. Nishida [65] proposed that nitrogenous fertilizers contribute to thickening the rice canopy, creating a humid and shaded microenvironment conducive to insect proliferation. Research by Bala et al. [51] revealed that the application of N alone or in high doses increased insect populations, whereas the application of P and K, either individually or in combination with N, decreased population growth. He also stated K confers strong resistance against insect pests, as high levels of K enhance secondary compound metabolism, decrease carbohydrate accumulation, and mitigate plant damage from insect pests. Pramanick et al. [66] found that fields receiving higher levels of K without or with minimal N experienced notably lower incidences of harmful insects. This aligns with our observation of reduced insect pest counts in PKS and KS applications (Fig. 4). Elevated K levels reduce food intake and assimilation by insects and offer strong resistance against insect pests by enhancing secondary compound metabolism, reducing carbohydrate accumulation, and minimizing plant damage caused by pests. Notably, a synergy between N and K levels was observed, with the most substantial enhancements in shoot and root dry matter occurring with increasing N levels at the highest K level. High K levels also led to decreased population growth and dry weight of BPH [53]. Excessive K amounts cause significant shifts in nutrients and allelochemicals, altering the plant's chemical environment and effectively suppressing insect populations. Additionally, K boosts resistance to insect pests by improving secondary compound metabolism and decreasing carbohydrate buildup, while excessive doses lead to decreased N uptake, negatively impacting insect biology and behavior. Crop plants accumulating high levels of K during favorable growth conditions may enhance their ability to withstand sudden environmental stresses [51]. Amtmann et al. [67] suggested that plants lacking K synthesis show decreased production of proteins, starch, and cellulose, while lighter-weight compounds such as amino acids, nitrate, soluble sugars, and organic acids increase. These lighter compounds become more accessible nutrient sources for piercing-sucking insects. Increased K fertilization of rice plants resulted in decreased populations of GLH, YSB, and RLR. Conversely, the infestation rate of YSB on rice plants was highest in the absence of K supply but declined with higher K concentrations [68]. In our study, P negatively influenced the incidence of harmful insects and natural enemy counts in the absence of N (Fig. 6). The population significantly decreased as the rate of P application increased. Phosphorus reduces the suitability of host plants against various insect pests by altering secondary metabolites such as phenolics and terpenes. Phenolics, including tannins and lignin, form a barrier with deterring or directly toxic effects on herbivores. Terpenes like monoterpenes, sesquiterpenes, and terpene polymers interfere with neural transmission, inhibit phosphorylation, and impede insect movement. Excessive dietary P at a 1 % concentration reduced the growth and survival of certain insects [69]. According to Skinner and Cohen [70], some pests may benefit from P-rich environments, while others, like the YSB, might suffer reduced performance. Conversely, Waring and Cobb [71] concluded that P either has no effect on sucking insects or may have a positive influence on them. Furthermore, Bala et al. [51] stated that P, Zn, and S reduce insect populations.

The MGIDI index identified NKS, NPS, and NPK fertilizer applications as EMTs in the rice field over two consecutive growing seasons, based on the performance of various parameters studied. Olivoto and Nardino [22] demonstrated in their experiments that the MGIDI is proficient in selecting genotypes based on multi-trait data, highlighting its usefulness in recommending treatments that excel across multiple parameters. Alam et al. [72] ranked 17 sweet potato genotypes based on the MGIDI index using multi trait performances and identified the strengths and weaknesses of ideal genotypes through factor analysis, supporting the methodology of the present study in identifying the EMT. Furthermore, a Venn diagram illustrating the MGIDI index over two years consistently identified NKS as the superior treatment for rice cultivation in BRRI, Habiganj. Alam et al. [72] employed a similar approach using a Venn diagram over two years to successfully identify high-yield and quality roots of sweet potato. The NKS treatment fulfilled the selection criteria, demonstrating its effectiveness in enhancing natural enemies in the rice field during both growing seasons, as evidenced by a positive SD for natural enemy counts. Moreover, a positive SD for rice grain yield during 2020-21 (0.37) was observed, further supporting the effectiveness of the selected EMT (NKS) in the weather conditions of Habiganj. Singamsetti et al. [32] also identified two maize hybrids that met the desired SDs across varying moisture conditions, indicating their potential for broader adaptation in rainfed environments prone to stress. This underscores their unique potential for wider use in such environments.

## 5 Conclusion

The findings from the two-year experiment conducted at the BRRI, Habiganj underscore the critical importance of effective fertilizer management in influencing insect prevalence and rice grain yield. Across both growing seasons, weather conditions during the mid-tillering and booting stages significantly impacted insect populations, with notable variations observed in harmful insects and their natural enemies. The relationship between these dynamics was strongly positive, highlighting the interdependence of insect populations and ecological balance in rice cultivation. The standard fertilizer dosage (NPKS) leads to the highest yield of rice grains, highlighting the efficacy of nitrogenous fertilizer in enhancing rice productivity. The use of nitrogenous fertilizer (NPKS, NKS, NPS and NPK) in rice farming is associated with an increase in the insect population within the rice field. Conversely, potash and phosphorus rich fertilizers, especially in the absence of nitrogen (KS and PS), result in a reduction of insect incidence. Among the eight tested chemical fertilizer applications, NKS consistently emerged as the most effective multidimensional treatment (EMT), characterized by its omission of phosphorus while containing nitrogen and potassium. NKS demonstrated significant positive effects on enhancing natural enemies and boosting rice grain yield over both years. Based on the findings, it is recommended to prioritize fertilizer treatment NKS, which excludes phosphorus but includes nitrogen and potassium, to optimize rice grain yield and promote natural enemy populations. This approach can contribute to minimizing harmful insect infestations while supporting ecological balance in rice fields. Furthermore, future research efforts should focus on refining fertilizer blends to achieve a harmonious balance between maximizing yield and fostering ecological robustness in rice cultivation. Investigating additional variables and factors that influence insect populations and natural enemies could provide further insights into optimizing fertilizer management practices for sustainable rice production. Adopting targeted fertilizer management strategies, such as emphasizing treatment NKS, holds promise for improving agricultural sustainability and resilience in rice cultivation, ultimately contributing to food security and ecological health.

## Data availability statement

Data will be made available on request.

## CRediT authorship contribution statement

**Sanjida Akter:** Writing – review & editing, Writing – original draft, Supervision, Software, Methodology, Investigation, Formal analysis, Data curation, Conceptualization. **Tapon Kumar Roy:** Formal analysis, Data curation. **Md Mozammel Haque:** Supervision, Resources, Investigation, Conceptualization. **Zakaria Alam:** Writing – review & editing, Visualization, Software, Formal analysis, Data curation.

## Declaration of competing interest

The authors declare that they have no known competing financial interests or personal relationships that could have appeared to influence the work reported in this paper.

## References

[1] Ito N. (2023). Hyper Low-cost Ultimate Rice Mechanization System: Sistema de Mecanização do Arroz Hyper Low-cost Ultimate. Braz. J. Anim. Environ. Res..

[2] Dey S., Badri J., Ram K., Chhabra A.K., Janghel D.K. (2019). Current status of rice breeding for sheath blight resistance. Int J Curr Microbiol Appl Sci.

[3] Rahman M.M., Hasan S., Ahmed MdR., Adham A.K.M. (2022). Recycling deep percolated water in continuously flooding irrigated rice fields to mitigate water scarcity. Paddy Water Environ..

[4] BBS, Summary Crop Statistics (2022).

[5] Kabir M.S., Salam M.U., Chowdhury A., Rahman N.M.F., Iftekharuddaula K.M., Rahman M.S., Rashid M.H., Dipti S.S., Islam A., Latif M.A. (2015). Rice vision for Bangladesh: 2050 and beyond, Bangladesh. Rice J..

[6] Timsina J., Panaullah G.M., Saleque M.A., Ishaque M., Pathan A.B.M.B.U., Quayyum M.A., Connor D.J., Saha P.K., Humphreys E., Meisner C.A. (2006). Nutrient uptake and apparent balances for rice-wheat sequences. I. Nitrogen. J. Plant Nutr..

[7] Sihi D., Dari B., Sharma D.K., Pathak H., Nain L., Sharma O.P. (2017). Evaluation of soil health in organic *vs* . conventional farming of basmati rice in North India. J. Plant Nutr. Soil Sci..

[8] Haque M.M., Biswas J.C., Islam M.R., Islam A., Kabir M.S. (2019). Effect of long-term chemical and organic fertilization on rice productivity, nutrient use-efficiency, and balance under a rice-fallow-rice system. J. Plant Nutr..

[9] Biswas J.C., Maniruzzaman M., Sattar M.A., Neogi M.G. (2008). Improvement of rice yield through fertilizer and cultural management at farmer's field. Bangladesh Rice J.

[10] Thurston H.D. (1984).

[11] Catling D. (2011). http://archive.saulibrary.edu.bd:8080/xmlui/bitstream/handle/123456789/4415/2010-2011-68.pdf?sequence=1.

[12] Ali M.P., Nessa B., Khatun M.T., Salam M.U., Kabir M.S. (2021). A way forward to combat insect pest in rice. Bangladesh Rice J..

[13] Magdoff F., Van Es H. (2000). https://www.soilandhealth.org/wp-content/uploads/0302hsted/030218bettersoils.pdf.

[14] Ye Z.Q. (2013). Accelerate the construction of modern plant protection with vigorously implements of green control. China Plant Prot.

[15] Donald C.M. (1968). The breeding of crop ideotypes. Euphytica.

[16] Rocha J.R.D.A.S.D.C., Machado J.C., Carneiro P.C.S. (2018). Multitrait index based on factor analysis and ideotype‐design: proposal and application on elephant grass breeding for bioenergy. GCB Bioenergy.

[17] Hazel L.N. (1943). The genetic basis for constructing selection indexes. Genetics.

[18] Smith H.F. (1936). A discriminant function for plant selection. Ann. Eugen..

[19] Barth E., de Resende J.T.V., Mariguele K.H., de Resende M.D.V., da Silva A.L.B.R., Ru S. (2022). Multivariate analysis methods improve the selection of strawberry genotypes with low cold requirement. Sci. Rep..

[20] Norman P.E., Agre P.A., Asiedu R., Asfaw A. (2022). Multiple-traits selection in White Guinea Yam (Dioscorea rotundata) genotypes. Plants.

[21] Rosero A., Burgos-Paz W., Araujo H., Pastrana-Vargas I.J., Martínez R., Pérez J.-L., Espitia L. (2023). Sweet potato varietal selection using combined methods of multi-trait index, genetic gain and stability from multi-environmental evaluations. Horticulturae.

[22] Olivoto T., Nardino M. (2021). MGIDI: toward an effective multivariate selection in biological experiments. Bioinformatics.

[23] Bizari E.H., Val B.H.P., Pereira E. de M., Mauro A.O.D., Unêda-Trevisoli S.H. (2017). Selection indices for agronomic traits in segregating populations of soybean1. Rev. Ciênc. Agronômica.

[24] Ahsan A.F.M.S., Alam Z., Ahmed F., Akter S., Khan M.A.H. (2024). Selection of waterlogging tolerant sesame genotypes (Sesamum indicum L.) from a dataset using the MGIDI index. Data Brief.

[25] Alam Z., Akter S., Khan M.A.H., Rashid M.H., Hossain M.I., Bashar A., Sarker U. (2023). Multi trait stability indexing and trait correlation from a dataset of sweet potato (Ipomoea batatas L.). Data Brief.

[26] Khan M.A.H., Rahim M.A., Robbani M., Hasan F., Molla M.R., Akter S., Ahsan A.F.M.S., Alam Z. (2024). Genotypic selection and trait variation in sweet orange (Citrus sinensis L. Osbeck) dataset of Bangladesh. Data Brief.

[27] Pimentel A.J.B., Guimarães J.F.R., de Souza M.A., de Resende M.D.V., Moura L.M., Rocha .R. do A.S. de C., Ribeiro G. (2014). Estimation of genetic parameters and prediction of additive genetic value for wheat by mixed models, Pesqui. Agropecuária Bras..

[28] Alam Z., Akter S., Khan M.A.H., Hossain M.I., Amin M.N., Biswas A., Rahaman E.H.M.S., Ali M.A., Chanda D., Rahman M.H.S. (2024). Sweet potato (Ipomoea batatas L.) genotype selection using advanced indices and statistical models: a multi-year approach. Heliyon.

[29] Kaiser H.F. (1958). The varimax criterion for analytic rotation in factor analysis. Psychometrika.

[30] Pour-Aboughadareh A., Poczai P. (2021). A dataset on multi-trait selection approaches for screening desirable wild relatives of wheat. Data Brief.

[31] Debsharma S.K., Syed M.A., Ali M.H., Maniruzzaman S., Roy P.R., Brestic M., Gaber A., Hossain A. (2022). Harnessing on genetic variability and diversity of rice (oryza sativa L.) genotypes based on quantitative and qualitative traits for desirable crossing materials. Genes.

[32] Singamsetti A., Zaidi P.H., Seetharam K., Vinayan M.T., Olivoto T., Mahato A., Madankar K., Kumar M., Shikha K. (2023). Genetic gains in tropical maize hybrids across moisture regimes with multi-trait-based index selection. Front. Plant Sci..

[33] Jalalifar R., Sabouri A., Mousanejad S., Dadras A.R. (2023). Estimation of genetic parameters and identification of leaf blast-resistant rice RILs using cluster analysis and MGIDI. Agronomy.

[34] Fao U. (1988).

[35] Ahmmed S., Jahiruddin M., Razia S., Begum R.A., Biswas J.C., Rahman A., Ali M.M., Islam K.M.S., Hossain M.M., Gani M.N. (2018). Fertilizer recommendation guide-2018. Bangladesh Agric. Res. Counc. BARC Farmgate Dhaka.

[36] BRRI (2022).

[37] Olivoto T., Lúcio A.D. (2020). metan: an R package for multi‐environment trial analysis. Methods Ecol. Evol..

[38] R studio R. (2020). https://www.r-project.org/.

[39] Jadhao M.F., Bhongade A.H. (2018).

[40] Gangurde S. (2007). Aboveground arthropod pest and predator diversity in irrigated rice (Oryza sativa L.) production systems of the Philippines. J. Trop. Agric..

[41] Kalita S., Hazarika L.K., Deka R.L., Gayon J. (2020). Effect of weather parameters and varieties on occurrence of insect pests and natural enemies of rice. J. Pharmacogn. Phytochem..

[42] Kumar S., Ram L., Kumar A. (2017). Population dynamics of white backed plant hopper, Sogatella furcifera on basmati rice in relation to biotic and weather parameters. J. Entomol. Zool. Stud..

[43] Kettle H., Sann C., Marion G. (2019). Quantifying parasitoid and predator controls on rice hopper eggs using a dynamic stage‐structured model and field data. J. Appl. Ecol..

[44] Horgan F.G., Srinivasan T.S., Naik B.S., Ramal A.F., Bernal C.C., Almazan M.L.P. (2016). Effects of nitrogen on egg-laying inhibition and ovicidal response in planthopper-resistant rice varieties. Crop Protect..

[45] Morshed M.N., Mamun M.A.A., Nihad S.A.I., Rahman M.M., Sultana N., Rahman M.M. (2023). Effect of weather variables on seasonal abundance of rice insects in southeast coastal region of Bangladesh. J. Agric. Food Res..

[46] Shepard B.M., Barrion A.T., Litsinger J.A. (1987).

[47] Moses S., Kishor D.R., Misra A.K., Ahmad M.A. (2019). Identification and quantification of major insect pests of rice and their natural enemies. Curr. J. Appl. Sci. Technol..

[48] Lu Z., Yu X., Heong K., Cui H.U. (2007). Effect of nitrogen fertilizer on herbivores and its stimulation to major insect pests in rice. Rice Sci..

[49] Zhu P., Zheng X., Xu H., Johnson A.C., Heong K.L., Gurr G.M., Lu Z. (2020). Nitrogen fertilizer promotes the rice pest Nilaparvata lugens via impaired natural enemy, Anagrus flaveolus, performance. J. Pest. Sci..

[50] de Kraker J. (1996). https://search.proquest.com/openview/1707d833737567f13a3630aecc035a9e/1?pq-origsite=gscholar&cbl=18750&diss=y.

[51] Bala K., Sood A.K., Pathania V.S., Thakur S. (2018). Effect of plant nutrition in insect pest management: a review. J. Pharmacogn. Phytochem..

[52] Chelliah S., Gunathilagaraj K. (2011). Pest management in rice-current status and future prospects. https://www.semanticscholar.org/paper/Pest-Management-in-Rice-Current-Status-and-Future-Chelliah-Gunathilagaraj/cbabbc66b70ad9822606b07145769931672d0486.

[53] Rashid M.M., Jahan M., Islam K.S. (2016). Impact of nitrogen, phosphorus and potassium on brown planthopper and tolerance of its host rice plants. Rice Sci..

[54] Kulagod S.D., Hegde M.G., Nayak G.V., Vastrad A.S., Hugar P.S. (2011). Influence of fertilizer on the incidence of insect pests in paddy. https://www.cabidigitallibrary.org/doi/full/10.5555/20113149306.

[55] Sun Y., Yuan X., Chen K., Wang H., Luo Y., Guo C., Wang Z., Shu C., Yang Y., Weng Y. (2023). Improving the yield and nitrogen use efficiency of hybrid rice through rational use of controlled-release nitrogen fertilizer and urea topdressing. Front. Plant Sci..

[56] Dukes J.S., Pontius J., Orwig D., Garnas J.R., Rodgers V.L., Brazee N., Cooke B., Theoharides K.A., Stange E.E., Harrington R., Ehrenfeld J., Gurevitch J., Lerdau M., Stinson K., Wick R., Ayres M. (2009). Responses of insect pests, pathogens, and invasive plant species to climate change in the forests of northeastern North America: what can we predict?This article is one of a selection of papers from NE Forests 2100: a Synthesis of Climate Change Impacts on Forests of the Northeastern US and Eastern Canada. Can. J. For. Res..

[57] Yamamura K., Yokozawa M. (2002). Prediction of a geographical shift in the prevalence of rice stripe virus disease transmitted by the small brown planthopper, Laodelphax striatellus (Fallen)(Hemiptera: Delphacidae), under global warming. Appl. Entomol. Zool..

[58] Svobodová E., Trnka M., Dubrovský M., Semerádová D., Eitzinger J., Štěpánek P., Žalud Z. (2014). Determination of areas with the most significant shift in persistence of pests in Europe under climate change. Pest Manag. Sci..

[59] Karthik S., Reddy M.S., Yashaswini G. (2021). Climate change and its potential impacts on insect-plant interactions, Nat. Causes Eff. Mitig. Clim. Change Environ..

[60] Shrestha S. (2019). Effects of climate change in agricultural insect pest. Acta Sci. Agric..

[61] Ram S., Patil B.D. (1986). Economic injury level of major defoliator insect pests of fodder cowpea, Vigna unguiculata (L.) Walp. J. Entomol. Res..

[62] Jian Y., Fu J., Wang X., Zhou F. (2023).

[63] Pandey S.K. (2003). Influence of different levels of nitrogen on the incidence of major insect pests of rice. J. Entomol. Res..

[64] Ghosh M.R. (1989). https://books.google.com/books?hl=en&lr=&id=IgjeORPkORwC&oi=fnd&pg=PA1&dq=ghosh+1962+nitrogen+insect&ots=_29c-W7jbM&sig=egC7g2f0FU9dP3UjOqXlqkt6G6E.

[65] Nishida T. (1975). Causes of brown planthopper outbreaks. Rice Entomol Newsl.

[66] Pramanick M., Das M., Ghosh M.R., Mukherjee N. (1995). Effect of fertilizer treatments on growth, productivity, insect-pest and disease incidences on rice. Madras Agric. J..

[67] Amtmann A., Troufflard S., Armengaud P. (2008). The effect of potassium nutrition on pest and disease resistance in plants. Physiol. Plantarum.

[68] Sarwar M. (2012). Effects of potassium fertilization on population build up of rice stem borers (lepidopteron pests) and rice (Oryza sativa L.) yield. J. Cereals Oilseeds.

[69] Facknath S., Lalljee B. (2005). Effect of soil‐applied complex fertiliser on an insect–host plant relationship: *Liriomyza trifolii* on *Solanum tuberosum*. Entomol. Exp. Appl..

[70] Skinner R.H., Cohen A.C. (1994). Phosphorus nutrition and leaf age effects on sweetpotato whitefly (Homoptera: aleyrodidae) host selection. Environ. Entomol..

[71] Waring G.L., Cobb N.S. (1992). The impact of plant stress on herbivore population dynamics. Insect-Plant Interact..

[72] Alam Z., Akter S., Khan M.A.H., Amin M.N., Karim M.R., Rahman M.H.S., Rashid M.H., Rahman M.M., Mokarroma N., Sabuz A.A., Alam M.J., Roy T.K., Rahaman E.H.M.S., Ali M.A., Chanda D., Sarker U. (2024). Multivariate analysis of yield and quality traits in sweet potato genotypes (Ipomoea batatas L.). Sci. Hortic..

